# Wildlife must be protected from crime and trade for the sake of public and planetary health

**DOI:** 10.1371/journal.pbio.3001422

**Published:** 2021-10-07

**Authors:** John E. Scanlon AO

**Affiliations:** Global Initiative to End Wildlife Crime, hosted by ADM Capital Foundation, Central, Hong Kong SAR, China

## Abstract

This Perspective article argues that as we struggle to combat climate change, with the potential loss of a million species and increasing exposure to thousands of new viruses that could spill over from wildlife to humans (as SARS-CoV-2 likely did), it is time to change course.

## Teaser

COVID-19 most likely had its origins in wildlife, and hundreds of thousands of new viruses could spill over from wildlife to humans. We are struggling to combat climate change, and we are staring down the loss of a million species. It’s time to change course.

We will, at some stage, get through the devastating Coronavirus Disease 2019 (COVID-19) pandemic. But when we do, it won’t be over. Far from it.

The most likely source of Severe Acute Respiratory Syndrome Coronavirus 2 (SARS-CoV-2) is a spillover from an animal to humans, possibly via another animal. While research is still ongoing to draw any final conclusions about the origins of COVID-19 [1], the links between wildlife and previous epidemics and pandemics, in particular of emerging infectious diseases (EIDs), are well known [2], as are the conditions that make the spillover of viruses from animals to humans more likely [3].

Health and wildlife experts have warned us for decades of the public health risks associated with people mixing with wild animals, including through habitat destruction, illegal or poorly regulated wildlife trade, and markets that bring together wild, captively bred, and domesticated animals [4]. It’s important to remember that, when left alone, wild animals pose no risk to human health: The risk comes from how we, as people, interact with wildlife.

In addition to these obvious health risks, the UN Intergovernmental Science-Policy Platform on Biodiversity and Ecosystem Services (IPBES) [5] predicts that, unless we change course, our actions including overexploitation and wildlife habitat destruction will drive over 1 million [6] species to extinction over the coming decades. The degradation of ecosystems will limit their ability to mitigate climate change, further fuelling the climate crisis. The COVID-19 pandemic led to a dramatic and sudden loss of revenue from wildlife tourism in 2020, which sent shock waves through the conservation community, decimating jobs, enterprises, and livelihoods. This loss of tourism [7] has, in turn, negatively affected the ability of countries to manage wildlife and protected areas, which is further exacerbating these threats to our biodiversity, climate, and public health. This pandemic has reminded us in a devastating way how interconnected our world is, most particularly between economies, the environment, human and wildlife health and welfare. We need to recalibrate our relationship with nature, which will require profound changes in how we regulate the taking, trade, and consumption of wildlife, how we combat wildlife crime, and how we manage and finance the protection of wildlife at its source.

I chair the Global Initiative to End Wildlife Crime, which is a diverse group of people and organisations coming from across every continent. Drawing upon the UN World Wildlife Crime Reports of 2016 [8] and 2020 [9], The World Bank Report of 2019 on Illegal Logging, Fishing, and Wildlife Trade: The Costs and How to Combat it [10], the 2019 IPBES Global Assessment Report on Biodiversity and Ecosystem Services [11], and the known links between trade in wild animals and the spillover of viruses to people, we have concluded that our current international legal framework for regulating wildlife trade and combating wildlife crime is inadequate both for regulating the trade, markets, and consumption that pose a risk to public health, as well as for ending wildlife crime.

Firstly, the international agreement that exists to regulate trade in wildlife, known by its acronym CITES (Convention on International Trade in Endangered Species of Wild Fauna and Flora), takes decisions based solely upon biological and trade criteria. It looks to see if trade will affect the survival of the species of animal or plant that is being traded, disregarding the risks that such trade could pose to human or animal health. This needs to change. Regulating wildlife trade needs to be based on a “One Health” approach, where biological, human health, and animal health criteria are all taken into consideration. Human memories are short, as is clear from the responses to previous pandemics, and to succeed, we must hardwire a “One Health” approach into the international legal framework. There are several viable options available to achieve this objective.

The most effective and cost-efficient way forward would be for States to adopt amendments to the CITES agreement that build human and animal health criteria into its decision-making processes and to extend its reach to wildlife markets. We know how to do this, and we have drafted a set of proposed amendments [12]. However, CITES Parties have traditionally been wary of extending its mandate, and they may elect not to take up this opportunity. Another option is to ensure that the scope of a proposed International Pandemics Treaty [13] includes legally binding commitments on taking measures to prevent the spillover of viruses and other pathogens from wild animals to people, including through wildlife trade and markets. We have released a short briefing paper [14] on this option, assessing the possible benefits of such a treaty.

Secondly, in addition to posing a threat to human and animal health, wildlife crimes are driving many species towards extinction, degrading entire ecosystems and their ability to sequester carbon, depriving governments of revenue, exacerbating corruption, insecurity, and poverty. If we include the impacts of these crimes on ecosystems, The World Bank [10] estimates their value at a staggering $1 to 2 trillion a year. Notwithstanding the destructive and high-risk nature of these crimes to both people and wildlife, there is no global agreement on wildlife crime, as there is, for example, on human trafficking. Humans and wildlife are losing, and the stark reality is that, left as it is, our system is not going to end these crimes or work to prevent the next pandemic.

We need to embed tackling wildlife crime into the international criminal law framework. On 17 May 2021, the President of Gabon, H. E. Ali Bongo Ondimba and the President of Costa Rica, H. E. Carlos Alvarado Quesada, jointly called to develop a new global agreement on wildlife crime that embeds preventing and combating it into the international criminal law framework, taking the form of a Fourth Protocol under the UN Convention Against Transnational Organised Crime. This visionary call was made by the Presidents of two countries that have been environmental leaders, countries that have led efforts to combat illicit trafficking of wild fauna and flora. Their call now needs to be supported by other States.

Wherever possible, it is best to take measures to stop the illegal taking of wild species, by better protecting them and their habitat, that is, protecting our last wild places, so wildlife is protected at its source. We can do that by focusing on long-term commitments to biodiverse rich, wild places, which are mainly found in developing countries.

The world is still feeling the full brunt of a pandemic that most likely had its origins in wildlife. We are advised that there are hundreds of thousands of new viruses that could spill over from wildlife to humans; we are struggling to combat climate change and staring down the loss of approximately 1 million species. Given the scale of the risks to people and the planet, we simply cannot stand by and watch wildlife continue to disappear without ratcheting up our collective response, including to our international wildlife laws and investment in wild places.

## Abbreviations

CITES: Convention on International Trade in Endangered Species of Wild Fauna and Flora
COVID-19: Coronavirus Disease 2019
EID: emerging infectious disease
IPBES: Intergovernmental Science-Policy Platform on Biodiversity and Ecosystem Services
SARS-CoV-2: Severe Acute Respiratory Syndrome Coronavirus 2
